# High-Resolution Mass Spectrometry-Based Metabolomics for Increased Grape Juice Metabolite Coverage

**DOI:** 10.3390/foods13010054

**Published:** 2023-12-22

**Authors:** Sébastien Nicolas, Benjamin Bois, Kevin Billet, Rémy Romanet, Florian Bahut, Jenny Uhl, Philippe Schmitt-Kopplin, Régis D. Gougeon

**Affiliations:** 1 Procédés Alimentaires et Microbiologiques, PAM UMR A 02.102, Université de Bourgogne-Institut Agro, Institut Universitaire de la Vigne et du Vin-Jules Guyot, F-21000 Dijon, France; sebastien.nicolas@u-bourgogne.fr (S.N.); kevin.billet@inrae.fr (K.B.); remy.romanet@sayens.fr (R.R.); florian.bahut@u-bourgogne.fr (F.B.); 2 Centre de Recherches de Climatologie, Biogéosciences UMR 6282, CNRS-Université de Bourgogne, Institut Universitaire de la Vigne et du Vin-Jules Guyot, F-21000 Dijon, France; benjamin.bois@u-bourgogne.fr; 3 DIVVA Platform, PAM UMR A 02.102, Institut Universitaire de la Vigne et du Vin-Jules Guyot, F-21000 Dijon, France; 4 Research Unit Analytical Biogeochemistry, Helmholtz Zentrum München, Ingolstaedter Landstrasse 1, 85764 Neuherberg, Germanyphilippe.schmittkopplin@helmholtz-munich.de (P.S.-K.); 5 Analytische Lebensmittel Chemie, Technische Universität München, Maximus-von-Imhof-Forum 2, 85354 Freising, Germany

**Keywords:** Chardonnay, Pinot noir, Meunier, Aligoté, molecular fingerprints, mesocarp

## Abstract

The composition of the juice from grape berries is at the basis of the definition of technological ripeness before harvest, historically evaluated from global sugar and acid contents. If many studies have contributed to the identification of other primary and secondary metabolites in whole berries, deepening knowledge about the chemical composition of the sole flesh of grape berries (i.e., without considering skins and seeds) at harvest is of primary interest when studying the enological potential of widespread grape varieties producing high-added-value wines. Here, we used non-targeted DI-FT-ICR-MS and RP-UHPLC-Q-ToF-MS analyses to explore the extent of metabolite coverage of up to 290 grape juices from four *Vitis vinifera* grape varieties, namely Chardonnay, Pinot noir, Meunier, and Aligoté, sampled at harvest from 91 vineyards in Europe and Argentina, over three successive vintages. SPE pretreatment of samples led to the identification of more than 4500 detected C,H,O,N,S-containing elemental compositions, likely associated with tens of thousands of distinct metabolites. We further revealed that a major part of this chemical diversity appears to be common to the different juices, as exemplified by Pinot noir and Chardonnay samples. However, it was possible to build significant models for the discrimination of Chardonnay from Pinot noir grape juices, and of Chardonnay from Aligoté grape juices, regardless of the geographical origin or the vintage. Therefore, this metabolomic approach opens access to a remarkable holistic molecular description of the instantaneous composition of such a biological matrix, which is the result of complex interplays among environmental, biochemical, and vine growing practices.

## 1. Introduction

Within the frame of winemaking, the composition and the analysis of grape juice—here considered as the sole flesh of berries—are of great importance throughout ripening up to harvest, as the juice contains primary metabolites, including sugars (notably glucose and fructose) and organic acids (notably L-tartaric and L-malic acids) [1], whose concentration evolution is at the basis of the decision to harvest [2]. Many studies have shown that their concentration at harvest is controlled by several factors, among which grape variety, vine growing practices, soil types, and environmental conditions with heat and water stress being at the center of the current concerns about climate change [2,3]. Amino acids also constitute major primary metabolites and fundamental nutrients for yeasts during alcoholic fermentation [4].

Grape juices also contain several known secondary metabolites, whose occurrence has also been the object of many studies. The major families include aroma compounds with C13-norisoprenoids, terpenes, benzene derivatives, alcohols, esters, and thiols, often present as glycosidic precursors [5,6,7,8,9,10,11,12]. Also included are phenolics with acid phenols in particular, peptides, carotenoids, and some flavonols, among which quercetin and hormones with abscisic acid (ABA) are a well know example, and vitamins [13,14,15,16,17]. A few thousand secondary metabolites have thus been observed and quantified so far in grape and wine matrices, thanks to various targeted analytical methodologies.

Furthermore, the last two decades have seen the advent of targeted and non-targeted metabolomic approaches, combined with advanced statistical tools, aiming at providing more comprehensive information about the actual chemical diversity, including low-concentrated metabolites, of various biological samples and body fluids [18,19]. In the case of grape and wine matrices, and as far as non-volatile compounds are concerned, Mass Spectrometry coupled to Liquid Chromatography (LC-MS) has certainly been central to the highest number of metabolomic analyses. LC-MS-based metabolomic analyses of grape berries have thus been applied to compare varieties, or to evaluate various impacts of vineyard practices or environmental parameters on the grape metabolome [20,21,22,23,24]. However, matrix effects are also central to LC-MS-based metabolomics, and particular attention has already been paid to sample preparation and to solvent extraction to get the best metabolite coverage in grape analysis [25,26]. These authors showed that a solvent containing approximately equal amounts of methanol and chloroform and up to 20% water allowed the detection of up to 4500 features by ElectroSpray Ionization Reverse Phase Ultra Performance Liquide Chromatography coupled with Time of Flight-Mass Spectrometry (ESI RP-UPLC-ToF-MS) of grape berries. However, as in several other studies, the different compartments of the berry (skin, flesh and seeds) were not analyzed separately.

Alternatively, the development of ultra-high resolution mass spectrometry with Direct Infusion Fourier Transform-Ion Cyclotron Resonance Mass Spectrometry (DI-FT-ICR-MS) has offered new perspectives for exploring the chemical complexity of bio/geo/chemical matrices [27]. Thanks to extraordinarily high sensitivity and mass resolution, DI-FT-ICR-MS non-targeted analyses of musts, wines, and spirits could, for example, provide unprecedented fingerprints [28], discriminate six varieties of grapes [29], identify forest-related specific fingerprints in barrel-aged wines [30], or simultaneously identify hundreds of volatile and non-volatile compounds in gin [31]. These analyses highlighted both the small amounts and the very minimal preparation steps required for remarkably efficient metabolite coverage in wine and spirit analysis. Although DI-FT-ICR-MS could be applied directly on methanol-diluted grape juice samples [32] thanks to the high dynamic range of the signals, grape juice fractionation by SPE is supposed to provide the highest metabolite coverage through the removal of highly abundant primary sugar metabolites, thus enhancing the detection of lower concentration secondary metabolites. In this context, combining DI-FT-ICR-MS with the highest resolution and thus compositional selectivity with the possibility to identify isobars by RP/HILIC-UHPLC-Q-ToF-MS^2^ has proven remarkably efficient for increasing the range of unknown detectable metabolites in life sciences and foods [33].

In this study, an extensive set of grape juices from two white grape varieties, *V. vinifera* Chardonnay and Aligoté, and two red grape varieties, Pinot noir and Meunier, collected at harvest in 13 different regions representing 91 vineyards in Europe and Argentina, were systematically analyzed in negative ionization mode by DI-FT-ICR-MS and RP-UHPLC-Q-TOF-MS in order to evaluate and compare the impact of two minimal sample preparations on the performance of metabolite coverage. These sample preparations were either diluted (non SPE), or SPE fractionated before analysis. We further used this dataset to probe the capacity of the combined methodologies to characterize the metabolite coverage and discriminate grape juices according to different parameters such as variety or geographical origin of the grape.

## 2. Materials and Methods

### 2.1. Grape Berry Collection

Grape juices were obtained throughout a random collection of berries from 91 distinct vineyards (Table 1), covering five countries (Argentina, France, Germany, Italy, and Portugal), 13 wine producing regions (Adige Valley, Alsace, Beaujolais, Bordeaux, Burgundy, Champagne, Douro, Languedoc, Piemont, Rheingau, Tarn, Uco Valley, and Württemberg), three vintages (2019, 2020, 2021) and four *Vitis vinifera* varieties (Aligoté, Chardonnay, Meunier, and Pinot noir). For each considered grape variety within a given plot and for a given vintage, grape bunches were randomly picked from incoming harvest cases throughout the harvest duration. To reflect the genuine natural conditions of the vineyards, we complied with the winegrowers’ decision to harvest (Table S1). Then, pseudo-biological replicates were made by randomly picking two pools of 100 berries within these grape bunches. In some cases (not enough berries), only one pool of 100 berries was used for a vineyard/vintage. The berries were then frozen to prevent any further development. Grape juices were finally obtained by manually pressing the thawed berries in plastic bags, and the collected juice was then frozen before further sample preparation (−20 °C).

### 2.2. Sample Preparation

Samples were defrosted at ambient temperature prior to vortexing and centrifugation (10 min, 10,000 rpm, 4 °C). The further preparation steps were according to Roullier-Gall, et al. [34]. To perform solid phase extraction (SPE), the samples’ supernatant was first acidified to pH2 with pure formic acid (LC-MS grade). Bond Elut C18 cartridges (100 mg, 1 mL, 120 μm, Agilent, Les Ulis, France) were then conditioned with 2 mL methanol (LC-MS grade), followed by 1 mL of acidified (pH2, formic acid) ultra-pure water (18.2 MΩ, Millipore, Merck, Darmstadt, Germany). Samples were then filtered through cartridges, which were washed with 1 mL of pH2 water prior to elution with 1 mL of methanol. All filtrations were performed using a CHROMABOND SPE vacuum manifold at −0.2 Bar. Before DI-FT-ICR-MS analysis, SPE extracts were 1/20 diluted in methanol. For RP-UHPLC-Q-TOF-MS, SPE extracts were 1/2 diluted in 5% acetonitrile. Non SPE preparation (direct dilution of the juices) was performed by diluting to 1/500 in methanol and to 1/5 in 5% acetonitrile for DI-FT-ICR-MS and RP-UHPLC-Q-ToF-MS, respectively.

### 2.3. DI-FT-ICR-MS 

Fourier Transform-Ion Cyclotron Resonance Mass Spectrometry (FT-ICR-MS) was used with direct infusion of samples to an Apollo II electrospray ionization (ESI) source, working in negative mode ESI (−), coupled to a 12T FT-ICR-MS (SolariX, Bruker Daltonics, Bremen, Germany). Mass spectra were acquired in negative ionization mode with a flow rate of 120 µL/h within a mass range of 92–1000 Da. A total of 400 scans were accumulated for each sample. Raw spectra were calibrated using Compass DataAnalysis 4.2 (Bruker Daltonics, Bremen, Germany), and peaks with a signal-to-noise ratio (S/N) above 3 were considered. The two matrices (SPE; non SPE) were then obtained by aligning all spectra of each type within a 0.5-ppm alignment error (defined as the ratio of the difference between two aligned masses (*m*/*z*_1_ − *m*/*z*_2_) to one of these masses (*m*/*z*_1_) × 10^6^). Molecular formulae were then assigned using an in-house developed software tool (NetCalc v2.0) [35].

### 2.4. RP-UHPLC-Q-ToF-MS

Ultra-High-Pressure Liquid Chromatography (UHPLC, Dionex Ultimate 3000, Thermo Fischer Scientific, Waltham, MA USA) was used for separation, coupled to a MaXis plus MQ ESI-Q-ToF mass spectrometer (Bruker, Bremen, Germany). Reverse Phase-Liquid Chromatography (RP-LC) was performed with an Acquity BEH C18 1.7 μm, 100 × 2.1 mm column (Waters, Guyancourt, France). Elution was performed using a gradient of water (A) and acetonitrile (B), both acidified with 0.1% (*v*/*v*) formic acid. For elution (40 °C), 5% (*v*/*v*) of eluent B was used from 0 to 1.1 min followed by a gradual increase of eluant B up to 99.5%, reached at 6.40 min. Detection was performed in negative ionization mode, within 100 to 1500 Da range, using an electrospray at two bars of pressure for the nebulizer and 10 L/min for nitrogen dry gas flow, an end plate offset of 500 V, and capillary voltage of 4500 V. To recalibrate spectra, four times diluted calibrant ESI-L Low Concentration Tuning Mix (Agilent, Les Ulis, France) was injected at the beginning of each run. Before batch analysis, the mass spectrometer was calibrated using undiluted Tuning Mix in enhanced quadratic mode, which allowed for the alignment of peaks with errors < 0.5 ppm. The two matrices (SPE; non SPE) were then obtained using the Metaboscape software (Bruker, Bremen, Germany, V 8.0.1), with the T-Rex 3D algorithm, using an intensity threshold of 4000 and considering [M−H]^−^, [M−H−H_2_0]^−^, [M+Cl−]^−^ ions.

### 2.5. Data Analysis

All statistical analyses and plot generation were performed within the R environment [36] (v 4.3.0). First, a data sanity check was performed over all datasets [37], leading to two samples being removed. Two other samples missing in one dataset (DI-FT-ICR-MS non SPE) were also removed from all datasets, leading to a total of 291 samples to be analyzed by each methodology. For data description, only features present in at least four samples were considered. As the four datasets (SPE, non SPE, for DI-FT-ICR-MS, and RP-UHPLC-Q-ToF-MS) were obtained separately, alignment among the datasets was performed as follows. The two DI-FT-ICR-MS datasets were aligned by matching the assigned formulas. For alignment using RP-UHPLC-Q-ToF-MS (UHPLC-Q-ToF-MS (SPE) vs. UHPLC-Q-ToF-MS (non SPE) or UHPLC-Q-ToF-MS vs. FT-ICR-MS), features identified in RP-UHPLC-Q-ToF-MS where first grouped within a 2-ppm alignment error window to identify potential isobars. The grouped *m*/*z* mean was then used to perform alignment with DI-FT-ICR-MS data within a 5-ppm alignment error mass range. For the RP-UHPLC-Q-ToF-MS vs. RP-UHPLC-Q-ToF-MS data alignment, a retention time (RT) tolerance of 10 s was used. In case of multiple matches, the lower mass differences and/or RT delta were selected. Prior to statistical analysis, batch effect was checked and corrected using the DBnorm package with the ber method [38,39]. Multivariate analysis was performed on features present in at least 33% of the samples, and any zero value was replaced with 2/3 of the minimum value for each feature [40]. Principal Component Analysis (PCA) and Orthogonal Partial Least Squares–Discriminant Analysis (OPLS–DA, n permutation = 500) were performed using ropls [37], after Log10 transformation and pareto scaling for all datasets. Redundancy Analysis (RDA) and Variance Partition were used to compare the impact of vintage, region, and cultivar [41,42] using the vegan package [43]. The significance of RDA models was tested with 1000 permutations. Univariate statistics were generated with the rstatix package [44]. Comparisons across treatment groups were performed using the Kruskal–Wallis test followed by Dunn’s *post-hoc* pairwise comparison. Intensities Fold Change (FC) between varieties were pairwise tested using either Student *t*-test or Mann–Whitney U-test. *p*-values were corrected (p.adjust) using the False Discovery Rate method (FDR) [45], and adjusted *p*-values < 0.05 were considered significant. To aid biological interpretation, the MetaCyc (v 26.0), Plant Metabolic Network (PMN, v 15.0), and GrapeCyc (v 9.0.1) databases were used to perform annotation on the identified ions of interest using the MetaboAnnotation package [46].

## 3. Results and Discussion

### 3.1. Global Metabolome of Grape Juices

**Figure 1:**
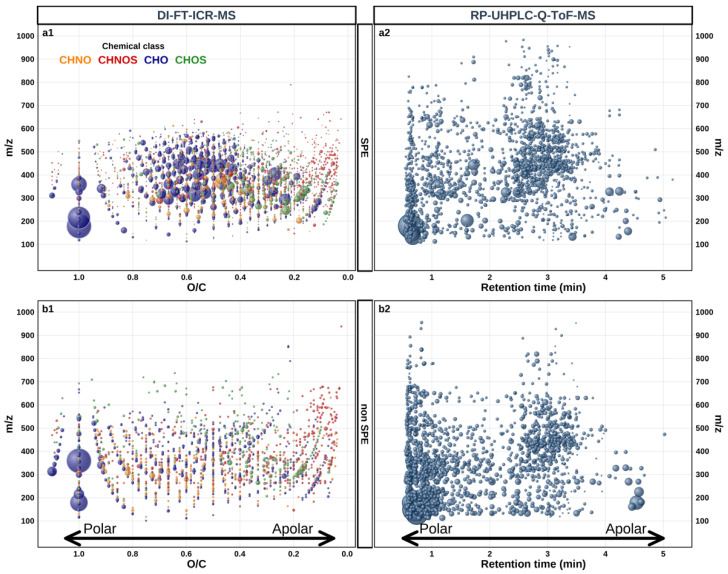
Display of the global grape juice metabolome detected by the different analytical strategies for SPE pretreated samples (**top**) and non SPE pretreated samples (**bottom**); (**a1**,**b1**) mass vs. O/C van Krevelen diagram representation of all detected DI-FT-ICR-MS mass peaks transformed into assigned elemental composition with an assignment error below 0.5 ppm, and a O/C ratio below 1.1. Dot sizes are proportional to the relative intensity of corresponding mass peaks, and with the following color codes: CHO (blue), CHON (orange), CHOS (green), CHONS (red); (**a2**,**b2**) all detected RP-UHPLC-Q-ToF-MS features represented as 2D maps projecting mass vs. RT (retention time).

The main objective of this study was to explore the extent of grape juice metabolite coverage, which can possibly be reached through integrated mass spectrometry-based metabolomics. In contrast with most LC-MS-based molecular analyses of grape berries [20,21,22,23,24,25,26], this study focused on the sole flesh of grape berries (mesocarp), i.e., without considering skins and seeds. To that purpose, up to 290 grape juice samples from four grape varieties (all characterized by uncolored flesh), were collected at harvest from 91 vineyards in Europe and Argentina (Table 1). The diversity in geographical origins was aimed at introducing the largest possible variability in compositions. Figure 1 gathers characteristic features resulting from the different analytical strategies considered here, when applied to all the samples, regardless of the geographical origin, the vintage, or the variety. The ESI (−) ionization mode was selected as a good compromise for the detection of the rather polar hydrophilic compounds consistently expected from acidic grape juices. In the case of DI-FT-ICR-MS, this mode has been shown to favor a larger variety in composition and abundance of compounds and a smaller number of adducts in wines, as well as a better resolution than positive ionization [47].

**Figure 2:**
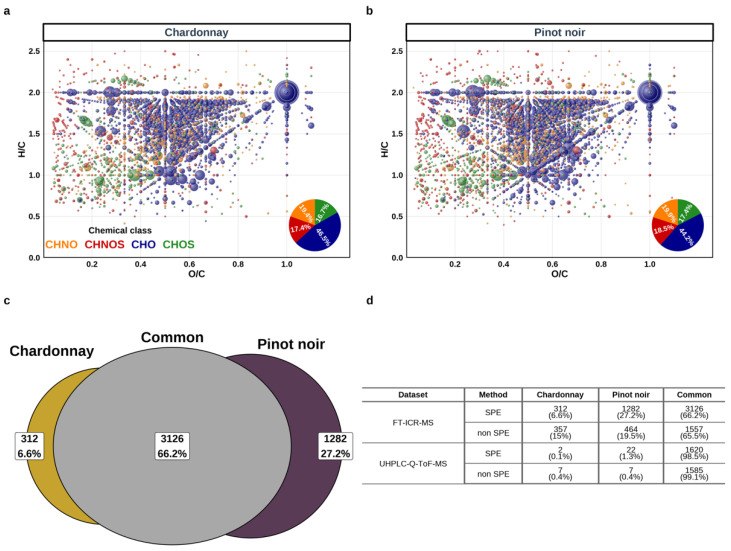
Comparison of the global Chardonnay and Pinot noir metabolomes obtained by DI-FT-ICR-MS, considering all geographical regions and vintages. H/C vs. O/C van Krevelen diagram representation for Chardonnay (**a**) and Pinot noir (**b**) with the following color codes for elemental compositions: CHO (blue), CHON (orange), CHOS (green), CHONS (red). These diagrams represent mass peaks annotated into assigned elemental composition with an assignment error below 0.5 ppm, and an O/C ratio below 1.1, a H/C ratio below 1.5, and present in at least four samples for each variety. Dot sizes are proportional to the relative intensity of corresponding mass peaks. Comparison between annotated mass peaks for the two cultivars is reported in a Venn Diagram (**c**). Summary of comparisons of numbers of detected features between the two cultivars for the four analytical methods of this study (**d**).

The ultra-high-resolution power of DI-FT-ICR-MS readily allowed the detection of up to 9000 mass signals with a signal-to-noise ratio >3 after SPE pretreatment of grape juices. Upon pre-processing these data, including mass difference network analysis, 4629 observed mass signals present in at least four samples could be converted into unambiguous elemental compositions based on the main isotopic elements (^12^C, ^14^N, ^16^O, ^32^S, ^1^H) within an assignment error window of 0.5-ppm (defined as the ratio of the difference between the experimental mass and the exact mass (*m*/*z*_exp_ − *m*/*z*_exact_) to the experimental mass (*m*/*z*_exp_) × 10^6^), thus providing an as yet unprecedented representation of the chemical diversity of the sole flesh of ripe grape berries (Figure 1(a1,b1), Figure 2a,b, Figures S1 and S2). Whether samples had been SPE pretreated or not, H/C vs. O/C van Krevelen diagrams (Figure 2a,b, Figures S1 and S2) revealed a remarkable metabolome coverage with likely tens of thousands of compounds (see below, Section 3.2). This metabolome, which comprises peptides and amino acids, fatty and organic acids [30,48], and extensive homologous series of conjugated compounds, i.e., alkylated compounds [49], and/or glycosylated compounds [50,51,52] consistently with such carbohydrate-rich matrices, provided a snapshot of the numerous metabolic pathways involved in the ripening of grape berries. Our results further revealed that common metabolites to SPE and non SPE pretreated samples could be found throughout the van Krevelen diagram regions, and covered a high range of polarity, suggesting that the hydrophilic character of FT-ICR-MS detected masses were weakly influenced by SPE adsorption behaviors, with likely high (−) ionization efficiency (Figure 1(a1,b1)). However, if both pretreatments led to the detection of metabolites over similar mass ranges (200–750 Da), the SPE pretreatment allowed for the detection of more abundant mass peaks between ca. 300 and 550 Da (Figure 1(a1)), characterized by medium polarity as expressed by their narrower range of O/C ratios, and mostly found in the peptide/nucleic acids and related conjugated compound region (Figure 2a,b and Figure S1). Such mass peaks would exhibit lower ESI (−) ionization efficiency and be suppressed otherwise without SPE pretreatment.

**Figure 3:**
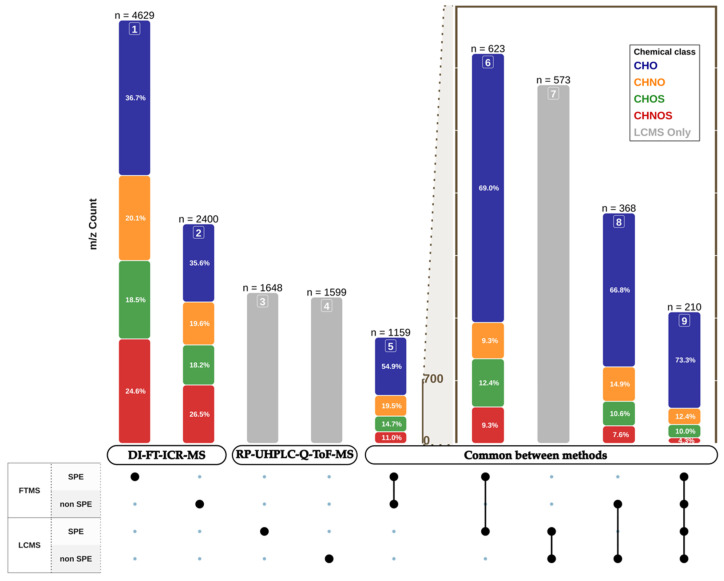
Histogram (upper part) of the number of unambiguously assigned elemental compositions based on DI-FT-ICR-MS (column 1–2) and RP-UHPLC-Q-ToF-MS (column 3–4) detected *m*/*z* values with the following color codes for elemental compositions: CHO (blue), CHNO (orange), CHOS (green), CHNOS (red). Single dots below associate elemental compositions to the pretreatment used for the two MS analyses, and connected dots indicate that these compositions are common to two or more pretreatments and analyses, after peak alignment (column 5–9).

Because of a significantly lower resolving power of RP-UHPLC-Q-ToF-MS, the 1648 and 1599 (Figure 3, Table S2) detected features for SPE and non SPE pretreatments, respectively, could not be straightforwardly and unambiguously transformed into elemental compositions, and the corresponding results were more rigorously presented as 2D maps projecting mass values as a function of retention times (Figure 1(a2,b2)). As confirmed by the density distributions of the detected mass peak intensity (Figure S2), the SPE pretreatment considerably reduced the number of hydrophilic low molecular weight metabolites (retention times < 1.5 min), thus favoring the detection of higher frequencies of less hydrophilic and heavier metabolites (mass > 250 Da, RT > 2.5 min), up to 1000 Da. Interestingly, some common buckets (RT; mass) to SPE and non SPE pretreated samples could be found at low retention times, indicating that SPE pretreatment did not remove all the highly hydrophilic metabolites initially present in the juice. However, it must be noted that under our experimental conditions (RP, C18 column), for simple grape juice dilution (non SPE) and to a lesser extent for SPE pretreated grape juices, the LC step did not prevent high abundance of detected mass peaks for retention times < 1 min (Figure 1(a2,b2) and Figure S2), which indicates that for low RTs, chromatographic separation could be considered inefficient, and which points to the need for HILIC-UHPLC-Q-ToF-MS for a further increase of the metabolite coverage [34].

Figure 2, which focuses on all the detected features in Chardonnay and Pinot noir samples only, provides a striking illustration of the high compositional similarity between grape juices of a red and a white wine grape variety. Considering the FT-ICR-MS results of SPE pretreated samples, 66.2% of the annotated mass peaks detected in at least four samples of Chardonnay and four samples of Pinot noir were common to the two varieties, whereas only 6.6% and 27.2% were only detected in Chardonnay and Pinot noir juices, respectively (Figure 2c). It must be noted that, although care had been taken to prevent juice/skin contact while Pinot noir berries were pressed, some skin extraction could possibly have occurred. This would contribute to the higher relative proportion of annotated masses specific to Pinot noir in the polyphenolic region of the van Krevelen diagram (H/C between 0.8 and 1.2; O/C around 0.5 and 0.7; Figure 2b). Figure 2d further gathers similar consideration for the other analytical methods, which shows that, under our experimental conditions, RP-UHPLC-Q-ToF-MS analyses of these grape juices could hardly detect features (present in at least four samples within each variety) specific to each of the varieties, with at best 22 out of a total of 1644, for SPE pretreated Pinot noir samples.

### 3.2. Aligning the Ultra-High-Resolution Power of DI-FT-ICR-MS with the UHPLC-Controlled High Resolution of RP-UHPLC-Q-ToF-MS

As shown in Figure 3, and in agreement with the literature [26,53], SPE pretreatment enabled the best metabolite coverage by DI-FT-ICR-MS with up to 4629 assigned elemental formulas, whereas for non SPE, only 2400 mass peaks could be assigned an elemental formula. This is a direct representation of the filtering impact of SPE pretreatment on ESI (−) DI-FT-ICR-MS leading to the significant reduction of adduct-formation salts, and of carbohydrates-in particular, sugars with concentrations at harvest being classically higher than 200 g/L-which contribute to a competition for ionization in the electrospray [54]. Figure 3 also indicates that whatever the pretreatment used (SPE or non SPE), up to 1159 mass peaks (and corresponding elemental compositions) were systematically detected in at least four samples, by DI-FT-ICR-MS. The same consideration for RP-UHPLC-Q-ToF-MS analyses led to 573 buckets (RT; mass) systematically found whatever the pretreatment used. This represented about a third of the number of buckets detected either by SPE (1648) or non SPE (1599) pretreated samples analyzed by RP-UHPLC-Q-ToF-MS. However, without alignment with DI-FT-ICR-MS data, it was unrealistic to assign elemental compositions to mass values related to these buckets, because of the lower resolving power of RP-UHPLC-Q-ToF-MS.

In previous papers [34], we have shown that the alignment of highly resolved mass peaks detected by DI-FT-ICR-MS with isobaric LC-separated mass peaks detected by LC-MS could clearly increase the scope of detectable unknown metabolites in wines. Despite a lower resolution, RP-UHPLC-Q-ToF-MS mass peaks with hits in DI-FT-ICR-MS peaks could then be assigned an unambiguous elemental formula (further validated by MS^2^), whereas multiple RP-UHPLC-Q-ToF-MS-detected retention times for a common mass peak would count the possible DI-FT-ICR-MS detected isobars. All results from all possible alignments were gathered in Table S2. A first striking feature was that after alignment within a 2-ppm alignment error window, distinct numbers of elemental compositions appeared to be common/unique to DI-FT-ICR-MS and RP-UHPLC-Q-ToF-MS analyses, depending on the aligned sets of data, illustrating the complementarity of the two platforms for grape juice analyses [34,47]. A maximum of 210 elemental compositions appeared to be common to the four analytical strategies (Figure 3, Table S2). Looking more closely at common compositions to DI-FT-ICR-MS and RP-UHPLC-Q-ToF-MS for non SPE pretreatment, Table 2 and Figure 3 show that 368 DI-FT-ICR-MS compositions (representing 12.2% of assigned compositions) found hits in RP-UHPLC-Q-ToF-MS spectra. Consistent with the filtering effect and the reduction of ion suppression of SPE, up to 623 hits (11.4%) were observed between the two MS methods, after SPE pretreatment. Since a given mass value observed in RP-UHPLC-Q-ToF-MS could be associated with multiple retention times, the count of RP-UHPLC-Q-ToF-MS hits could be higher than for DI-FT-ICR-MS, as shown both for SPE and non SPE pretreated samples, with up to 1035 (62.8%) and 825 (51.6%) aligned peaks, respectively.

If the average number of observed retention times was between one and two for a given mass value, Table 2 further showed that some DI-FT-ICR-MS mass peaks could frequently be associated with up to four retention times, and in some case up to 13 distinct retention times. Assuming a restrictive 2-ppm alignment error window for the alignment procedure within RP-UHPLC-Q-ToF-MS spectra, and considering that only about 12% of the DI-FT-ICR-MS peaks found hits in RP-UHPLC-Q-ToF-MS peaks, one could hypothesize that the actual chemical diversity probed by DI-FT-ICR-MS in SPE treated grape juice samples is certainly of a few tens of thousands of metabolites. Furthermore, upon breaking down common mass peaks into distinct compositions, Table 2 showed that most of the mass peaks aligned between the two MS methods were assigned CHO-based elemental formulas. This result highlighted the complementary power of DI-FT-ICR-MS to more evenly probe the diversity of S/N-containing metabolites (Figure 3). It must be noted that the case of 13 distinct retention times was observed for non SPE samples (Table 2), for a mass peak at 215.03279 Da easily assigned the C_6_H_12_O_6_ [M+Cl]^−^ ion formula, corresponding to a chlorine adduct of glucose [55]. The observation of up to 13 retention times over the 1–5.74 min range in RP-UHPLC-Q-ToF-MS spectra raised the question of competition for ionization in non SPE pretreated samples, with the possible need to better adjust the dilution. An explanation would be that highly abundant glucose and fructose molecules, considered here as ”pollutants”, are eluted throughout the chromatographic time frame.

### 3.3. Grape Juice Discrimination by ESI (−) DI-FT-ICR-MS Applied to SPE Pretreated Samples

With such a high dimensionality of the data set, both in terms of individuals (grape juice samples, Table 1) and variables (assigned elemental compositions, Figure 3), it is possible to explore the ability of metabolomics to discriminate specific molecular fingerprints among subsets of samples. Of the four grape varieties considered in this study, Chardonnay and Pinot noir were the most widely represented in terms of vintages and geographical origins and therefore terroirs, thus providing an unprecedented array of likely subtle variations in metabolite expressions. However, if the discrimination between red grape (Pinot noir) and white grape (Chardonnay) cultivars can be straightforward when considering skin extracts because of the higher polyphenol concentrations for the former, discrimination based on the sole berry juices, regardless of both the geographical origin and the vintage, remains challenging, as demonstrated by Figure 2. As shown by a variance partition study (Figure S3) of a data subset corresponding to DI-FT-ICR-MS analyses (SPE pretreatment) of Chardonnay and Pinot noir grape juices for vintages 2019 and 2020, the variety and the geographical origin could significantly explain only 15.9% and 10.4% of the variance, respectively, whereas nearly 75% of the variance appeared unexplained by these parameters or the vintage. Furthermore, the Redundancy Analysis (RDA) of mass peaks, which significantly contribute to the geographical origin-related variance, showed that the Languedoc region would be more different from Burgundy than the Southern Hemisphere region of the Uco Valley in Argentina. These results (Figure S3) emphasized that, for such a diversity of grape juice origins, environmental conditions along with vineyard practices can indeed contribute to significantly modulating metabolite expressions even within a single grape variety, thus introducing possibly significant noise into targeted analyses. In contrast, non-targeted analyses can provide comprehensive transient chemical snapshots of highly subtle metabolite expressions, which are considered to integrate contributions from every plant–environment interaction associated with the multiple vineyard conditions, and provided that the sampling is large enough, it is possible to apply robust multivariate statistical analyses to such a dataset.

**Figure 4:**
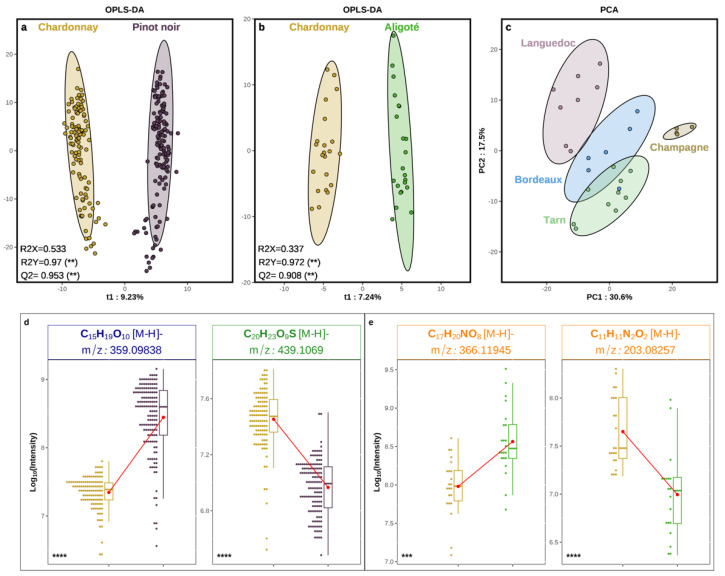
Multivariate statistical analyses of DI-FT-ICR-MS metabolomic analyses of SPE pretreated samples, regardless of the geographical origin or the vintage; supervised OPLS-DA discrimination of Chardonnay/Pinot noir grape juices (**a**) and Chardonnay/Aligoté grape juices (**b**); non supervised PCA analysis of Pinot noir and Meunier grape juices (**c**); each dot in (**a**–**c**) refers to a grape juice sample; jitter plots and boxplots showing examples of VIP *m*/*z* peaks for Chardonnay and Pinot noir (**d**) and for Chardonnay and Aligoté (**e**). Each jitter plot provides a density distribution of the corresponding mass peak intensity (expressed as the Log_10_ value) among Chardonnay (yellow) and Pinot noir (purple) samples (**d**), and among Chardonnay (yellow) and Aligoté (green) samples (**e**); **, *** and **** indicate significance with *p* value at 1E−2, 1E−3, and 1E−4, respectively.

Figure 4a,b show that OPLS-DA models could significantly discriminate Chardonnay from Pinot noir grape juices, and Chardonnay from Aligoté grape juices (two white cultivars), respectively. In both cases, the robustness of the model was guaranteed by Q2-values > 0.9, for the quality of prevision, and R2Y-values > 0.97, for the goodness of the fit. Furthermore, similar significant discriminations could also be obtained with non SPE pretreated samples, and again with whatever the sample pretreatment used (SPE/non SPE) with RP-UHPLC-Q-ToF-MS (Table S3). In contrast, attempts to discriminate between the two red cultivars (Pinot noir and Meunier) failed through cross-validation steps, and the non-supervised PCA statistical analysis appeared to be controlled by the geographical origins of the vineyards regardless of the grape variety, with the first two components explaining more than 48% of the variance (Figure 4c). A much smaller set of samples (with only four geographical origins and two vintages, Table 1) could possibly contribute to this model failure for the two red grape cultivars, but interestingly, when reduced to the same geographical origins as those for Meunier, data subsets for Pinot noir and Chardonnay could still lead to a significantly discriminant model (Table S3). This result suggests that Pinot noir and Meunier grape juices could be too similar, and their discrimination would be primarily driven by vineyard characteristics, with Champagne (emblematic land of Meunier) being best discriminated (Figure 4c).

The molecular fingerprints for both Pinot noir and Chardonnay were dominated by CHO compositions with up to 80.7% (142 assigned elemental formulas) and 50.9% (31 assigned elemental formulas) of Variable Importance in Projection (VIPs), respectively (Figure S4). SPE pretreated Pinot noir grape juices clearly appeared much richer in glycosylated homologous series of polyphenolic structures (Figure S2). Although care had been taken to prevent juice/skin contact while Pinot noir berries were pressed, some skin extraction could not be completely ruled out, and these homologous series would possibly be partly attributed to skin metabolites. Interestingly, when considering VIPs which discriminated among non SPE treated samples (Figure S5), CHO compositions were still dominating fingerprints, but with significantly fewer mass values (13 CHO elemental compositions representing 59.1% for Pinot noir, and 26 CHO compositions representing 43.3% for Chardonnay). However, up to 35% of Chardonnay VIPs were S-containing compositions, including 15 CHNOS (25%) and 6 CHOS (10%), which confirmed the relatively high importance of N/S-containing metabolites for this cultivar.

Finally, Figure 3 reports examples of VIP elemental compositions among tens of others and likely hundreds of related compounds (Table S4), with the 359.09838 *m*/*z* peak, to which the [C_19_H_19_O_10_]^−^ ion composition could be assigned for Pinot noir, and the 439.1068 *m*/*z* peak, to which the [C_20_H_23_O_9_S]^−^ ion composition could be assigned for Chardonnay. The latter S-containing formula was better observed as part of a three-membered homologous series with an O/C ratio of around 0.4 in non SPE pretreated samples (Figure S4) but no pertinent structural assignment could be found in accessible databases. In contrast, the CHO marker for Pinot noir, could likely correspond to a glycosidic adduct of syringic acid or isomers, which have been identified in other *Vitis vinifera* red grapes [56], and whose concentration in berries could be modulated by vine growing management. As witnessed by the four associated RT from RP-UHPLC-Q-ToF-MS results, up to four glycosidic isomers could possibly be more abundant in Pinot noir grape juices, depending for instance on the O-glycosylation position. As to examples of VIPs discriminant for Chardonnay and Aligoté, Figure 3 shows that the 203.08257 *m*/*z* peak, to which the [C_11_H_11_N_2_O_2_]^−^ ion composition can be assigned, was significantly more abundant in Chardonnay grape juices, whereas the 366.11945 *m*/*z* peak, to which the [C_17_H_20_NO_8_]^−^ ion composition can be assigned, was significantly more abundant in Aligoté. A consistent structural assignment to the Chardonnay VIP could be Tryptophan, as this grape variety has been shown to be among the most concentrated in this precursor of indoleacetic acid [57,58]. Interestingly, the Aligoté marker shown in Figure 3 could correspond to indolelactic acid glucoside, another glucoside already identified in white grapes as a contributor to the “phenolic taste” of white wines [59]. This would be the first identification of this metabolite in the rarely studied Aligoté grape variety.

## 4. Conclusions

In this study, a large series of juices of ripe grape berries from four different varieties, sampled in various vineyards internationally and over three successive vintages, were analyzed by DI-FT-ICR-MS and RP-UHPLC-Q-ToF-MS, to explore the possible extent of metabolome coverage. Samples were either SPE pretreated or not, before analysis. DI-FT-ICR-MS analyses of SPE pretreated samples clearly provided higher metabolite coverage, with only about 13% of the 4629 assigned elemental compositions being common to RP-UHPLC-Q-ToF-MS detected masses. Our results revealed that the sole flesh of Chardonnay, Pinot noir, Meunier, and Aligoté berries could likely contain tens of thousands of compounds transiently present throughout the ripening period up to harvest. This result is even more remarkable given that such chemical diversity does not include the many seed- and skin-related metabolites such as polyphenols. Additionally, when considering Chardonnay and Pinot noir, the two most represented grape varieties in our sampling, we have shown that up to 75% of this chemical diversity is common to all juices, thus emphasizing the fact that many similar metabolic pathways must be involved in the ripening of these two varieties. Only 15.9% of the variance of Pinot noir and Chardonnay metabolomes appeared to be explained by the variety. However, thanks to the high dimensionality of our sampling and of the detected metabolomes, it was possible to build significant models for the discrimination of Chardonnay from Pinot noir grape juices, and of Chardonnay from Aligoté grape juices, regardless of the geographical origin or the vintage. Several tens of related chemical markers could be identified, including, for example, glycosides for Pinot noir. In summary, an excellent complementarity between the two analytical methods was shown, with FT-ICR-MS being a rapid, reproducible, and highly precise tool for non-targeted sample screening, and RP-UHPLC-Q-ToF-MS complementing ideally with the possible resolution of isomers through the chromatographic dimension, and the MS/MS fragmentation tools for structural identifications. Such results are of primary importance when studying the enological potential of emblematic grape varieties such as Pinot noir and Chardonnay, which can produce high-added-value wines from many different vineyards around the world.

## Figures and Tables

**Table 1:** Details of the number of samples collected for each variety in function of regions and vintages.

Region	Aligoté			Chardonnay			Meunier			Pinot Noir		
	2019	2020	2021	2019	2020	2021	2019	2020	2021	2019	2020	2021
Adige valley										10		
Alsace										2	4	4
Beaujolais				1								
Bordeaux	1		2	1	2	2	1		2	1	2	2
Burgundy	4	4	2	20	35	8				29	26	10
Champagne				2		1	1		1	2		1
Douro				4								
Gaillac	1	2	2	1	2	2	1	2	2	1	2	2
Languedoc	2	2		6	6		2	2		6	6	
Piedmont				2	2					2	3	
Rheingau										14		
Uco Valley				5	6					8	6	
Württemberg				2			2					

**Table 2:** Focus on the number of features related to DI-FT-ICR-MS and RP-UHPLC-Q-ToF-MS mass peak alignments for SPE and non SPE pretreatments.

Method	DI-FT-ICR-MS			RP-UHPLC-Q-ToF-MS			
	n (Total)	Chemical Class	n Chemical Class	n Total ^1^	n Chemical Class ^1^	n Isobars	
						Mean ^2^	Range ^2^
SPE	623 (11.4%)	CHO	430 (69%)	1035 (62.8%)	794 (76.7%)	1.5	1–6
		CHNO	58 (9.3%)		71 (6.9%)	1.1	1–3
		CHNOS	58 (9.3%)		77 (7.4%)	1.2	1–6
		CHOS	77 (12.4%)		93 (9%)	1.1	1–4
non SPE	368 (12.2%)	CHO	246 (66.8%)	825 (51.6%)	623 (75.5%)	1.8	1–13
		CHNO	55 (14.9%)		99 (12%)	1.4	1–4
		CHNOS	28 (7.6%)		47 (5.7%)	1.3	1–4
		CHOS	39 (10.6%)		56 (6.8%)	1.2	1–3

^1^ “n” corresponds to the number of common elemental compositions between the two analytical methods within a 2-ppm mass peak alignment error. ^2^ “n isobaric mean” is the average number of retention times associated with a given elemental composition and “n isobaric range” indicates the range of retention times associated with a given elemental composition (RP-UHPLC-Q-ToF-MS analyses).

## Data Availability

Data is contained within the article or Supplementary Materials.

## Supplementary Materials

The following supporting information can be downloaded at: https://www.mdpi.com/article/10.3390/foods13010054/s1, Figure S1: H/C vs. O/C van Krevelen diagrams representing all detected DI-FT-ICR-MS mass peaks transformed into assigned elemental compositions, Figure S2: Display of the global grape juice metabolome detected by the different analytical strategies, Figure S3: Multivariate statistical analysis of Chardonnay and Pinot noir grape juice metabolomes, detected by DI-FT-ICR-MS after SPE pretreatment, Figure S4: S-plot and van Krevelen diagrams of SPE prepared samples (Chardonnay and Pinot noir grape juices) analyzed by DI-FT-ICR-MS, Figure S5: S-plot and van Krevelen diagrams of non SPE prepared samples (Chardonnay and Pinot noir grape juices) analyzed by DI-FT-ICR-MS, Table S1: Summary of the harvest date ranges for the different vintages, varieties and hemispheres, Table S2: Summary of all DI-FT-ICR-MS and RP-UHPLC-Q-ToF-MS mass peaks alignments broken down by common elemental compositions, Table S3: Statistical analyses parameters for the different grape variety discriminations shown in Figure 4, Table S4: Summary of all DI-FT-ICR-MS and RP-UHPLC-Q-ToF-MS mass peak alignments that significatively discriminate Chardonnay and Pinot noir.
