# Supplementary material for: High-Resolution Mass Spectrometry-Based Metabolomics for Increased Grape Juice Metabolite Coverage
Source: Foods. 2023 Dec 22;13(1):54. doi: 10.3390/foods13010054 (PMC10778666; doi:10.3390/foods13010054)
Supplement: Supplementary file 1 [file foods-13-00054-s001.zip › FigureSup_S1.pdf]

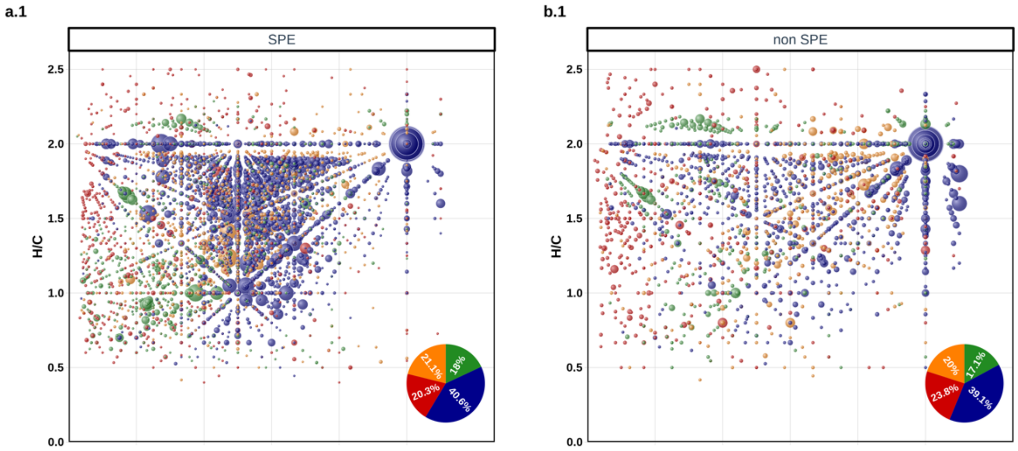

Figure S1: H/C vs O/C van Krevelen diagrams representation of all detected DI-FT-ICR-MS mass peaks transformed into assigned elemental compositions for SPE pretreated samples (left) and non SPE pretreated samples (right) with the following color code for elemental compositions: CHO (blue), CHON (orange), CHOS (green), CHONS (red)
