# Supplementary material for: High-Resolution Mass Spectrometry-Based Metabolomics for Increased Grape Juice Metabolite Coverage
Source: Foods. 2023 Dec 22;13(1):54. doi: 10.3390/foods13010054 (PMC10778666; doi:10.3390/foods13010054)
Supplement: Supplementary file 1 [file foods-13-00054-s001.zip › FigureSup_S2.pdf]

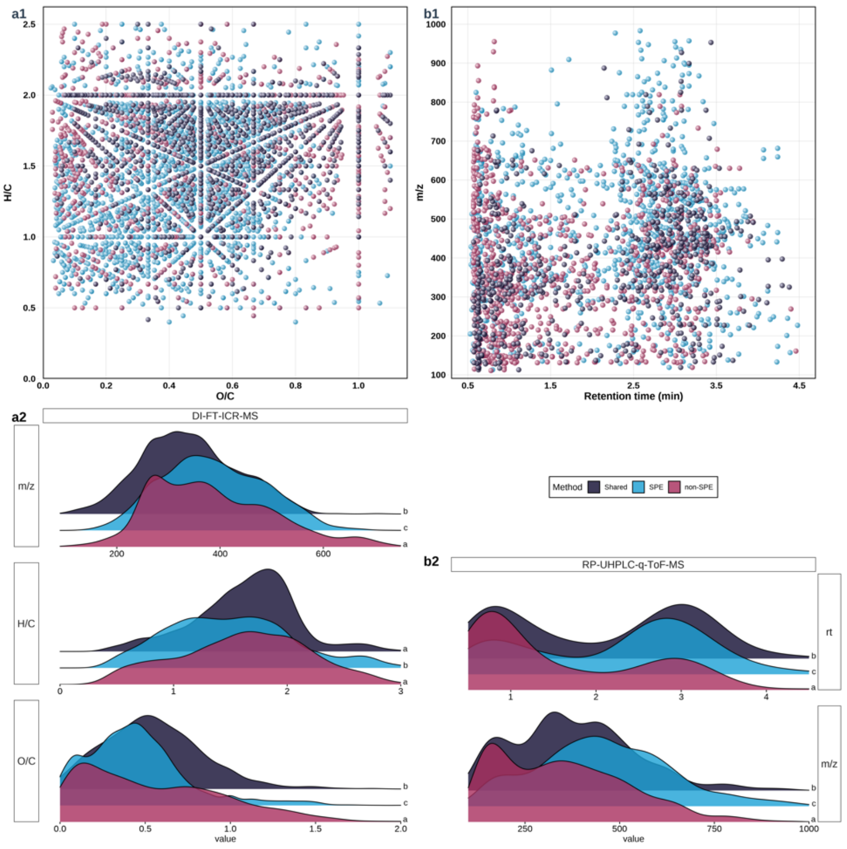

Figure S2: Display of the global grape juice metabolome detected by the different analytical strategies, with features unique to SPE pretreated samples (blue), non SPE pretreated samples (red) and common to SPE and non SPE (black); (a1) H/C vs O/C van Krevelen diagrams representation of DI-FT-ICR-MS mass peaks transformed into assigned elemental compositions; (b1) 2D- map projecting RP-UHPLC-Q-ToF-MS features (RT vs mass); (a2) Density distribution of mass peak intensities of DI-FT-ICR-MS mass peaks expressed as H/C elemental compositions (top), as O/C elemental compositions (middle) and as m/z values (bottom); (b2) Density distribution of mass peak intensities of RP-UHPLC-Q-ToF-MS features as a function of RT (top), m/z values (bottom).
