# Supplementary material for: High-Resolution Mass Spectrometry-Based Metabolomics for Increased Grape Juice Metabolite Coverage
Source: Foods. 2023 Dec 22;13(1):54. doi: 10.3390/foods13010054 (PMC10778666; doi:10.3390/foods13010054)
Supplement: Supplementary file 1 [file foods-13-00054-s001.zip › FigureSup_S3.pdf]

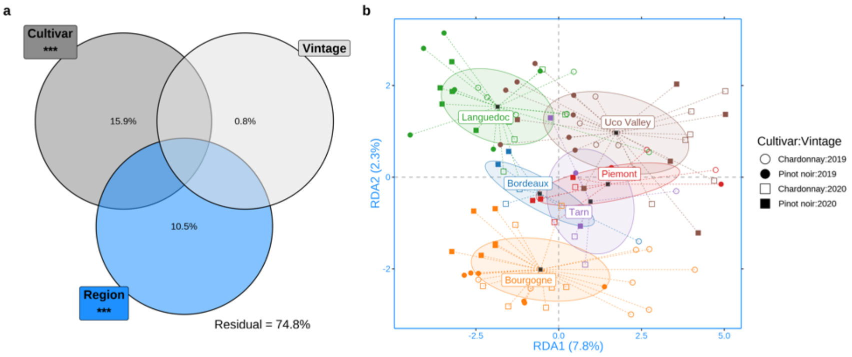


c

| Year | Cultivar | Bordeaux | Bourgogne | Languedoc | Piemont | Tarn | Uco Valley |
|---|---|---|---|---|---|---|---|
| 2019 | CH | 1 | 8 | 6 | 2 | 1 | 5 |
| | PN | 1 | 6 | 6 | 2 | 1 | 8 |
| 2020 | CH | 2 | 7 | 6 | 2 | 2 | 6 |
| | PN | 2 | 6 | 6 | 3 | 2 | 6 |

**Figure S3:** Multivariate statistical analysis of Chardonnay and Pinot noir grape juice metabolomes, detected by DI-FT-ICR-MS after SPE pretreatment; (a) Venn diagram representation of the variance partition of the entire dataset; (b) Redundancy Analysis (RDA) of the metabolome subset, which contributes to the variance explained by the geographical origin of juices, with the first two components (10.1%) contributing to nearly all the variance presented in (a), *i.e* 10.5%; (c) summary of samples used for the variance partition and RDA studies.
