# Supplementary material for: High-Resolution Mass Spectrometry-Based Metabolomics for Increased Grape Juice Metabolite Coverage
Source: Foods. 2023 Dec 22;13(1):54. doi: 10.3390/foods13010054 (PMC10778666; doi:10.3390/foods13010054)
Supplement: Supplementary file 1 [file foods-13-00054-s001.zip › FigureSup_S4.pdf]

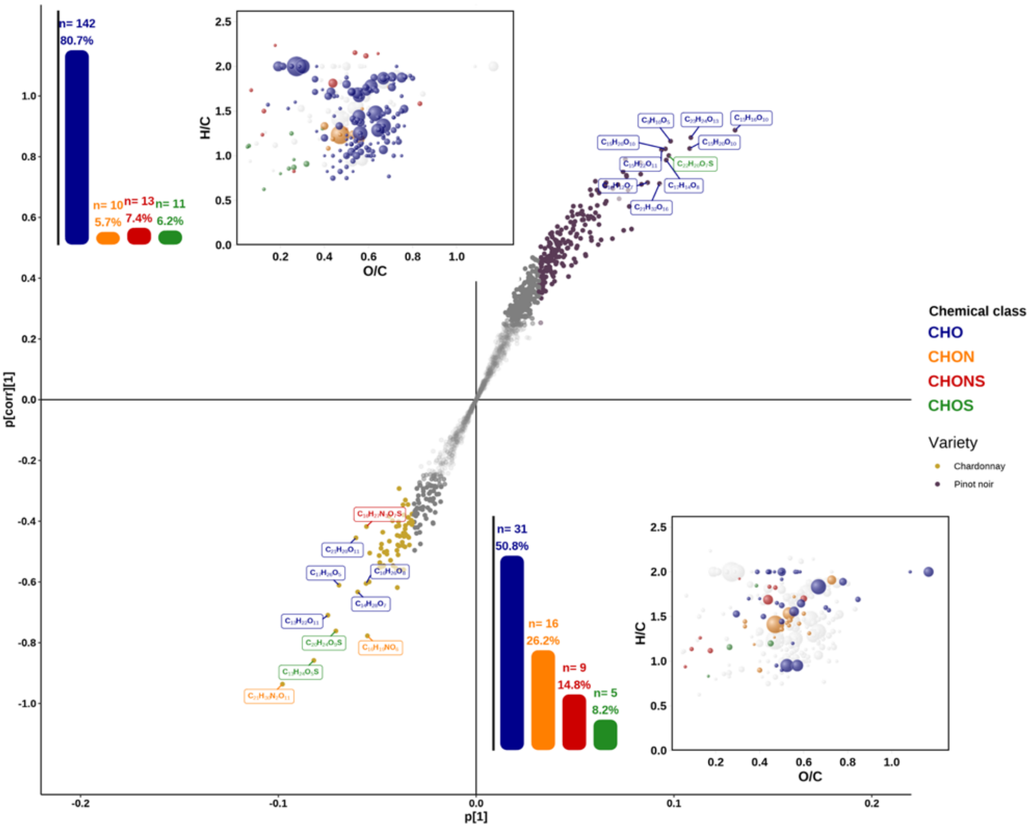

Figure S4: S-plot and van Krevelen diagrams of SPE prepared samples (Chardonnay and Pinot noir grape juices) analyzed by FT-ICR-MS. For the Splot, points are colored if OPLS-DA VIP values are > 1.3 (fold change tests), and the color is associated to the grape variety for which formulas in the van Krevelen diagam are more concentrated. For van Krevelen diagrams and associated histograms, the color code is associated to the chemical class (CHO (blue), CHNO (orange), CHOS (green), CHNOS (red)). Only VIPs up regulated in Pinot noir (upper part) or in Chardonnay (lower part) are highlighted and light grey dots represent masses down regulated for this variety.
