# Supplementary material for: High-Resolution Mass Spectrometry-Based Metabolomics for Increased Grape Juice Metabolite Coverage
Source: Foods. 2023 Dec 22;13(1):54. doi: 10.3390/foods13010054 (PMC10778666; doi:10.3390/foods13010054)
Supplement: Supplementary file 1 [file foods-13-00054-s001.zip › SupTable.pdf]

Table S1. Summary of the harvest date ranges for the different vintages, varieties and hemispheres.

Vintage	Hemisphere	Variety	Harvest dates range (min ; max)
2019	North	Aligoté	2019-08-27 ; 2019-09-18
		Chardonnay	2019-08-22 ; 2019-09-24
		Meunier	2019-08-27 ; 2019-09-16
		Pinot noir	2019-08-27 ; 2019-10-01
	South	Chardonnay	2019-03-06 ; 2019-03-20
		Pinot noir	2019-02-28 ; 2019-03-20
2020	North	Aligoté	2020-08-14 ; 2020-09-10
		Chardonnay	2020-08-14 ; 2020-09-11
		Meunier	2020-08-14 ; 2020-08-20
		Pinot noir	2020-08-14 ; 2020-09-11
	South	Chardonnay	2020-02-20 ; 2020-03-09
		Pinot noir	2020-02-21 ; 2020-03-06
2021	North	Aligoté	2021-08-31 ; 2021-09-27
		Chardonnay	2021-08-31 ; 2021-10-14
		Meunier	2021-08-31 ; 2021-10-14
		Pinot noir	2020-08-25 ; 2021-10-14

Table S2. Summary of all DI-FT-ICR-MS and RP-UHPLC-Q-ToF-MS mass peaks alignments broken down by common elemental compositions. The color code for common elemental compositions is the same as in Figure 3, "n" corresponds to the number of common elemental compositions between the two analytical methods within a 2 ppm mass peaks alignment error, "mean" is the average number of retention times associated with a given elemental composition (LC-MS analyses), and "range" indicates the range of retention times associated with a given elemental composition (LC-MS analyses). The column ID refer to the column number in Figure 3.

ID	DI-FT-ICR-MS			RP-UHPLC-Q-ToF-MS		DI-FT-ICR-MS		RP-UHPLC-Q-ToF-MS SPE				RP-UHPLC-Q-ToF-MS non SPE			
	Chemical Class	SPE	non SPE	SPE	non SPE	n Total	n Chemical Class	n Total	n Chemical Class	n isobares mean	n isobares range	n Total	n Chemical Class	n isobares mean	n isobares range
1	CHO	✓				4 629	1 700 (36.7%)								
	CHNO						931 (20.1%)								
	CHNOS						1 141 (24.6%)								
	CHOS						857 (18.5%)								
2	CHO		✓			2 400	855 (35.6%)								
	CHNO						470 (19.6%)								
	CHNOS						637 (26.5%)								
	CHOS						438 (18.2%)								
3				✓				1,648							
4					✓							1,599			
5	CHO	✓	✓			1 159 (SPE : 21.1%) (non SPE : 38.6%)	636 (54.9%)								
	CHNO						226 (19.5%)								
	CHNOS						127 (11%)								
	CHOS						170 (14.7%)								
6	CHO	✓		✓		623 (11.4%)	430 (69%)	1035 (62.8%)	794 (76.7%)	1.8	1--8				
	CHNO						58 (9.3%)		71 (6.9%)	1.2	1—5				
	CHNOS						58 (9.3%)		77 (6.9%)	1.3	1--6				
	CHOS						77 (12.4%)		93 (9%)	1.2	1--4				
7				✓	✓			573 (34.8%)				573 (35.8%)			
8	CHO		✓		✓	368 (12.2%)	246 (66.8%)					825 (51.6%)	623 (75.5%)	2.5	1--13
	CHNO						55 (14.9%)						99 (12%)	1.8	1--5
	CHNOS						28 (7.6%)						47 (5.7%)	1.7	1--6
	CHOS						39 (10.6%)						56 (6.8%)	1.4	1--4

ID	DI-FT-ICR-MS			RP-UHPLC-Q-ToF-MS		DI-FT-ICR-MS		RP-UHPLC-Q-ToF-MS SPE				RP-UHPLC-Q-ToF-MS non SPE			
	Chemical Class	SPE	non SPE	SPE	non SPE	n Total	n Chemical Class	n Total	n Chemical Class	n isobares		n Total	n Chemical Class	n isobares	
										mean	range			mean	range
9	CHO	✓	✓	✓	✓	210 (SPE : 3.8%) (non SPE : 7%)	154 (73.3%)	294 (28.3.4%)	234 (79.6%)	1.5	1--6	294 (37.5%)	234 (79.6%)	1.5	1--6
	CHNO						26 (12.4%)		29 (9.9%)	1.1	1--2		29 (9.9%)	1.1	1--2
	CHNOS						9 (4.3%)		10 (3.4%)	1.1	1--2		10 (3.4%)	1.1	1--2
	CHOS						21 (10%)		21 (7.1%)	1.0	1--1		21 (7.1%)	1.0	1--1

Table S3. Statistical analyses parameters for the different grape variety discriminations shown in Figure 4; CH : Chardonnay, PN : Pinot noir , Al : Aligoté, Me : Meunier. Subset[1] : subset of samples from similar vineyards as in CH~Al subset; Subset[2] : subset of samples from similar vineyards as in PN~Me subset.

Measure Method	Samples preparation	Comparaison	n samples	n features	R2X(cum)	R2Y(cum)	Q2(cum)	RMSEE	n orthogonal	p-value R2Y	p-value Q2
DI-FT-ICR-MS	SPE	CH~PN	252	1677	0.533	0.97	0.953	0.0863	4	0.002	0.002
	non SPE			599	0.535	0.975	0.944	0.0793	5	0.002	0.002
RP-UHPLC-Q-ToF-MS	SPE			1449	0.369	0.984	0.977	0.064	2	0.002	0.002
	non SPE			1283	0.278	0.979	0.958	0.0733	3	0.002	0.002
DI-FT-ICR-MS	SPE	CH~Al	44	1379	0.337	0.972	0.908	0.0873	2	0.002	0.002
	non SPE			702	0.5	0.986	0.837	0.0641	4	0.002	0.002
RP-UHPLC-Q-ToF-MS	SPE			1412	0.321	0.993	0.949	0.0437	2	0.002	0.002
	non SPE			1240	0.204	0.989	0.673	0.0552	2	0.002	0.002
DI-FT-ICR-MS	SPE	CH~PN Subset[1]	44	1551	0.48	0.982	0.839	0.0718	3	0.002	0.002
	non SPE			706	0.532	0.988	0.851	0.0596	4	0.002	0.002
RP-UHPLC-Q-ToF-MS	SPE			1476	0.415	0.988	0.941	0.0576	2	0.002	0.002
	non SPE			1282	0.265	0.989	0.824	0.0555	2	0.002	0.002
DI-FT-ICR-MS	SPE	CH~PN Subset[2]	28	1622	0.509	0.983	0.779	0.072	3	0.002	0.002
	non SPE			786	0.573	0.994	0.76	0.0424	4	0.002	0.002
RP-UHPLC-Q-ToF-MS	SPE			1482	0.441	0.992	0.925	0.0475	2	0.002	0.002
	non SPE			1289	0.297	0.996	0.798	0.0331	2	0.002	0.002
DI-FT-ICR-MS	SPE	PN~Me	28	1693							
	non SPE			707							
RP-UHPLC-Q-ToF-MS	SPE			1467							
	non SPE			1467							

Table S4. Summary of all DI-FT-ICR-MS and RP-UHPLC-Q-ToF-MS mass peaks alignments that significatively discriminate Chardonnay and Pinot noir. All the masses have a VIP value >1.3 and an adjusted p.vale (FDR) <0.05 for the test of comparaison of intensity means test comparaison. m/z values refer to the negative ion mass; rt: retention time; n : number of retention times for a given mass.

Method	DI-FT-ICR-MS						RP-UHPLC-Q-ToF-MS				Data base Annotation		
	m/z	Formula	Adduct	Class	H/C	O/C	m/z	rt	Ions	n	Name	Data base ID	Data base
SPE	293.1241615	$C_{12}H_{22}O_8$	[M-H]-	CHO	1.833	0.667	293.12377	2.41	[M-H]-	2			
							293.12390	2.52	[M-H]-	2			
	307.1762035	$C_{14}H_{28}O_7$	[M-H]-	CHO	2.000	0.500	307.17570	1.15	[M-H]-	2			
							307.17566	1.33	[M-H]-	2			
	315.0510072	$C_{16}H_{12}O_7$	[M-H]-	CHO	0.750	0.438	315.05062	2.95	[M-H]-	2	1,5,7-trihydroxy-6,8-dimethoxyanthraquinone	CPD-9630	PlantCyclMetaCyc
											3-O-methylquercetin	3457-TETRAHYDROXY-3-METHOXYFLAVONE	PlantCyclMetaCyc
											8-methoxykaempferol	CPD-16764	PlantCyclMetaCyc
											demethylsulochrin	DESMETHYLSULOCHRIN	MetaCyc
											isorhamnetin	CPD-8004	GrapeCyclPlantCyclMetaCyc
											petunidin	CPD-15003	MetaCyc
											rhamnetin	CPD-13511	PlantCyclMetaCyc
											robustaquinone E	CPD-9619	PlantCyclMetaCyc
											selgin	CPD-12571	PlantCyclMetaCyc
											tamarixetin	CPD-20494	MetaCyc
							315.05043	4.12	[M-H]-	2	1,5,7-trihydroxy-6,8-dimethoxyanthraquinone	CPD-9630	PlantCyclMetaCyc
											3-O-methylquercetin	3457-TETRAHYDROXY-3-METHOXYFLAVONE	PlantCyclMetaCyc
											8-methoxykaempferol	CPD-16764	PlantCyclMetaCyc
											demethylsulochrin	DESMETHYLSULOCHRIN	MetaCyc
											isorhamnetin	CPD-8004	GrapeCyclPlantCyclMetaCyc
											petunidin	CPD-15003	MetaCyc
											rhamnetin	CPD-13511	PlantCyclMetaCyc
											robustaquinone E	CPD-9619	PlantCyclMetaCyc
											selgin	CPD-12571	PlantCyclMetaCyc
											tamarixetin	CPD-20494	MetaCyc
	333.0615928	$C_{16}H_{14}O_8$	[M-H]-	CHO	0.875	0.500	333.06094	3.07	[M-H]-	2	8-O-methyl-13-carboxynorjavanicin	CPD-18190	MetaCyc
											8-O-methyl-fusarubinlactone	CPD-18197	MetaCyc
							333.06067	3.22	[M-H]-	2	8-O-methyl-13-carboxynorjavanicin	CPD-18190	MetaCyc
											8-O-methyl-fusarubinlactone	CPD-18197	MetaCyc
	355.0670738	$C_{15}H_{16}O_{10}$	[M-H]-	CHO	1.067	0.667	355.06676	1.17	[M-H]-	3			
							355.06674	2.56	[M-H]-	3			
	359.0983807	$C_{15}H_{20}O_{10}$	[M-H]-	CHO	1.333	0.667	359.09796	1.11	[M-H]-	4			
							359.09797	1.31	[M-H]-	4			
							359.09789	1.51	[M-H]-	4			

Method	DI-FT-ICR-MS						RP-UHPLC-Q-ToF-MS				Data base Annotation		
	m/z	Formula	Adduct	Class	H/C	O/C	m/z	rt	Ions	n	Name	Data base ID	Data base
							359.09804	1.83	[M-H]-	4			
	367.1610000	$C_{15}H_{28}O_{10}$	[M-H]-	CHO	1.867	0.667	367.16046	1.73	[M-H]-	2	(2S)-((6-O-(β-D-apiofuranosyl)β-D-glucopyranosyl)oxy)butane	CPD-15071	MetaCyc
							367.16037	2.15	[M-H]-, [M+Cl]-	2	(2S)-((6-O-(β-D-apiofuranosyl)β-D-glucopyranosyl)oxy)butane	CPD-15071	MetaCyc
	393.1766500	$C_{17}H_{30}O_{10}$	[M-H]-	CHO	1.765	0.588	393.17581	2.50	[M-H]-	2			
							393.17543	2.99	[M-H]-	2			
	403.1610097	$C_{18}H_{28}O_{10}$	[M-H]-	CHO	1.556	0.556	403.16042	2.86	[M-H]-	2			
							403.16017	3.02	[M-H]-	2			
	413.1664951	$C_{16}H_{30}O_{12}$	[M-H]-	CHO	1.875	0.750	413.16613	1.74	[M-H]-	2			
							413.16592	2.16	[M-H]-	2			
	427.0858000	$C_{22}H_{20}O_7S$	[M-H]-	CHOS	0.909	0.318	427.08511	1.11	[M-H]-	2			
							427.08498	1.50	[M-H]-	2			
	435.2236000	$C_{20}H_{36}O_{10}$	[M-H]-	CHO	1.800	0.500	435.22286	2.67	[M-H]-	2			
							435.22254	3.05	[M-H]-	2			
	477.1039597	$C_{22}H_{22}O_{12}$	[M-H]-	CHO	1.000	0.545	477.10288	2.60	[M-H]-	2	isorhamnetin 3-O-glucoside	CPD-14969	PlantCyclMetaCyc
											isorhamnetin 7-O-glucoside	CPD-14972	MetaCyc
											rhamnetin 3-O-glucoside	CPD-14970	MetaCyc
							477.10287	3.04	[M-H]-	2	isorhamnetin 3-O-glucoside	CPD-14969	PlantCyclMetaCyc
											isorhamnetin 7-O-glucoside	CPD-14972	MetaCyc
											rhamnetin 3-O-glucoside	CPD-14970	MetaCyc
	495.1145000	$C_{22}H_{24}O_{13}$	[M-H]-	CHO	1.091	0.591	495.11381	2.79	[M-H]-	2			
							495.11360	2.90	[M-H]-	2			
	507.1146000	$C_{23}H_{24}O_{13}$	[M-H]-	CHO	1.043	0.565	507.11419	2.53	[M-H]-	4	eupatolitin 3-glucoside	CPD-14911	PlantCyclMetaCyc
							507.11386	2.86	[M-H]-	4	eupatolitin 3-glucoside	CPD-14911	PlantCyclMetaCyc
							507.11367	3.04	[M-H]-	4	eupatolitin 3-glucoside	CPD-14911	PlantCyclMetaCyc
							507.11404	3.20	[M-H]-	4	eupatolitin 3-glucoside	CPD-14911	PlantCyclMetaCyc
	509.1302000	$C_{23}H_{26}O_{13}$	[M-H]-	CHO	1.130	0.565	509.12926	1.81	[M-H]-	3			
							509.12967	2.58	[M-H]-, [M-H-H2O]-	3			
							509.12934	2.82	[M-H]-	3			
	509.2241000	$C_{22}H_{38}O_{13}$	[M-H]-	CHO	1.727	0.591	509.22330	2.74	[M-H]-	3	2,4-di(3-methylbutanoyl)sucrose	CPD-19120	PlantCyclMetaCyc
											2,4-di-(2-methyl)butanoyl-sucrose	CPD-20291	MetaCyc
											2-(2-methyl)butanoyl-4-isovaleroyl-sucrose	CPD-20292	MetaCyc
											3',4-di-(3-methyl)butanoyl-sucrose	CPD-19613	MetaCyc
											3,4-di(3-methylbutanoyl)sucrose	CPD-21955	PlantCyclMetaCyc
											3-(2-methylbutanoyl)-4-(3-methylbutanoyl)sucrose	CPD-19025	PlantCyclMetaCyc
							509.22347	3.05	[M-H]-	3	2,4-di(3-methylbutanoyl)sucrose	CPD-19120	PlantCyclMetaCyc
											2,4-di-(2-methyl)butanoyl-sucrose	CPD-20291	MetaCyc
											2-(2-methyl)butanoyl-4-isovaleroyl-sucrose	CPD-20292	MetaCyc
											3',4-di-(3-methyl)butanoyl-sucrose	CPD-19613	MetaCyc
											3,4-di(3-methylbutanoyl)sucrose	CPD-21955	PlantCyclMetaCyc
											3-(2-methylbutanoyl)-4-(3-methylbutanoyl)sucrose	CPD-19025	PlantCyclMetaCyc

Method	DI-FT-ICR-MS						RP-UHPLC-Q-ToF-MS				Data base Annotation		
	m/z	Formula	Adduct	Class	H/C	O/C	m/z	rt	Ions	n	Name	Data base ID	Data base
							509.22352	3.21	[M-H]-	3	2,4-di(3-methylbutanoyl)sucrose	CPD-19120	PlantCyclMetaCyc
											2,4-di-(2-methyl)butanoyl-sucrose	CPD-20291	MetaCyc
											2-(2-methyl)butanoyl-4-isovaleroyl-sucrose	CPD-20292	MetaCyc
											3',4-di-(3-methyl)butanoyl-sucrose	CPD-19613	MetaCyc
											3,4-di(3-methylbutanoyl)sucrose	CPD-21955	PlantCyclMetaCyc
											3-(2-methylbutanoyl)-4-(3-methylbutanoyl)sucrose	CPD-19025	PlantCyclMetaCyc
	517.1505000	$C_{14}H_{30}N_8O_9S_2$	[M-H]-	CHNOS	2.143	0.643	517.15063	2.86	[M-H]-	3			
							517.14982	3.08	[M-H]-	3			
							517.14981	3.30	[M-H]-	3			
	567.2297000	$C_{24}H_{40}O_{15}$	[M-H]-	CHO	1.667	0.625	567.22722	2.86	[M-H]-	2			
							567.22653	3.03	[M-H]-	2			
	641.1364000	$C_{27}H_{30}O_{18}$	[M-H]-	CHO	1.111	0.667	641.13464	2.27	[M-H]-	2	myricetin 3-O-gentiobioside	CPD-14844	PlantCyclMetaCyc
							641.13494	2.82	[M-H]-	2	myricetin 3-O-gentiobioside	CPD-14844	PlantCyclMetaCyc
non SPE	315.1085297	$C_{14}H_{20}O_8$	[M-H]-	CHO	1.429	0.571	315.10843	1.23	[M-H]-	3			
			[M-H]-	CHO	1.429	0.571	315.10825	2.54	[M-H]-	3			
			[M-H]-	CHO	1.429	0.571	315.10819	2.76	[M-H]-	3			
	329.0877987	$C_{14}H_{18}O_9$	[M-H]-	CHO	1.286	0.643	329.08718	1.05	[M-H]-	4	1-O-vanilloyl-β-D-glucose	CPD-15006	GrapeCyclPlantCyclMetaCyc
			[M-H]-	CHO	1.286	0.643	329.08739	1.74	[M-H]-	4	1-O-vanilloyl-β-D-glucose	CPD-15006	GrapeCyclPlantCyclMetaCyc
			[M-H]-	CHO	1.286	0.643	329.08731	2.00	[M-H]-	4	1-O-vanilloyl-β-D-glucose	CPD-15006	GrapeCyclPlantCyclMetaCyc
			[M-H]-	CHO	1.286	0.643	329.08729	2.71	[M-H]-	4	1-O-vanilloyl-β-D-glucose	CPD-15006	GrapeCyclPlantCyclMetaCyc
	345.0827273	$C_{14}H_{18}O_{10}$	[M-H]-	CHO	1.286	0.714	345.08262	0.92	[M-H]-	2			
			[M-H]-	CHO	1.286	0.714	345.08275	1.13	[M-H]-	2			
	355.1034696	$C_{16}H_{20}O_9$	[M-H]-	CHO	1.250	0.562	355.10305	2.63	[M-H]-	2	1-O-feruloyl-β-D-glucose	CPD-15281	PlantCyclMetaCyc
			[M-H]-	CHO	1.250	0.562	355.10288	2.92	[M-H]-	2	1-O-feruloyl-β-D-glucose	CPD-15281	PlantCyclMetaCyc
	359.0984756	$C_{15}H_{20}O_{10}$	[M-H]-	CHO	1.333	0.667	359.09819	1.40	[M-H]-	5			
			[M-H]-	CHO	1.333	0.667	359.09805	1.65	[M-H]-	5			
			[M-H]-	CHO	1.333	0.667	359.09791	2.36	[M-H]-	5			
			[M-H]-	CHO	1.333	0.667	359.09798	2.55	[M-H]-	5			
			[M-H]-	CHO	1.333	0.667	359.09793	2.77	[M-H]-	5			
	447.0933779	$C_{21}H_{20}O_{11}$	[M-H]-	CHO	0.952	0.524	447.09274	3.23	[M-H]-	2	aureusidin 6-O-glucoside	CPD-8634	GrapeCyclPlantCyclMetaCyc
			[M-H]-	CHO	0.952	0.524	447.09274	3.23	[M-H]-	2	cyanidin 5-O-β-D-glucoside	CPD-7386	PlantCyc
			[M-H]-	CHO	0.952	0.524	447.09274	3.23	[M-H]-	2	cyanidin-3-O-β-D-galactoside	CPD-14998	PlantCyclMetaCyc
			[M-H]-	CHO	0.952	0.524	447.09274	3.23	[M-H]-	2	cyanidin-3-O-β-D-glucoside	CPD1F-766	GrapeCyclPlantCyc
			[M-H]-	CHO	0.952	0.524	447.09274	3.23	[M-H]-	2	isoorientin	ISOORIENTIN	PlantCyclMetaCyc
			[M-H]-	CHO	0.952	0.524	447.09274	3.23	[M-H]-	2	kaempferol 3-O-β-D-galactoside	CPD-7260	PlantCyclMetaCyc
			[M-H]-	CHO	0.952	0.524	447.09274	3.23	[M-H]-	2	kaempferol 3-O-β-D-glucoside	CPD1F-453	GrapeCyclPlantCyclMetaCyc
			[M-H]-	CHO	0.952	0.524	447.09274	3.23	[M-H]-	2	kaempferol 7-O-glucoside	CPD-8007	PlantCyc
			[M-H]-	CHO	0.952	0.524	447.09274	3.23	[M-H]-	2	luteolin 4'-O-β-D-glucoside	CPD-25365	MetaCyc
			[M-H]-	CHO	0.952	0.524	447.09274	3.23	[M-H]-	2	luteolin 7-O-β-D-glucoside	LUTEOLIN-7-O-BETA-D-GLUCOSIDE	PlantCyclMetaCyc
			[M-H]-	CHO	0.952	0.524	447.09274	3.23	[M-H]-	2	orientin	CPD-15083	PlantCyclMetaCyc
			[M-H]-	CHO	0.952	0.524	447.09274	3.23	[M-H]-	2	quercetin 3-O-rhamnoside	CPD-8013	PlantCyclMetaCyc
			[M-H]-	CHO	0.952	0.524	447.09274	3.23	[M-H]-	2	quercitrin	QUERCITRIN	MetaCyc

Method	DI-FT-ICR-MS						RP-UHPLC-Q-ToF-MS				Data base Annotation		
	m/z	Formula	Adduct	Class	H/C	O/C	m/z	rt	Ions	n	Name	Data base ID	Data base
			[M-H]-	CHO	0.952	0.524	447.09286	3.31	[M-H]-	2	aureusidin 6-O-glucoside	CPD-8634	GrapeCyc|PlantCyc|MetaCyc
			[M-H]-	CHO	0.952	0.524	447.09286	3.31	[M-H]-	2	cyanidin 5-O-β-D-glucoside	CPD-7386	PlantCyc
			[M-H]-	CHO	0.952	0.524	447.09286	3.31	[M-H]-	2	cyanidin-3-O-β-D-galactoside	CPD-14998	PlantCyc|MetaCyc
			[M-H]-	CHO	0.952	0.524	447.09286	3.31	[M-H]-	2	cyanidin-3-O-β-D-glucoside	CPD1F-766	GrapeCyc|PlantCyc
			[M-H]-	CHO	0.952	0.524	447.09286	3.31	[M-H]-	2	isoorientin	ISOORIENTIN	PlantCyc|MetaCyc
			[M-H]-	CHO	0.952	0.524	447.09286	3.31	[M-H]-	2	kaempferol 3-O-β-D-galactoside	CPD-7260	PlantCyc|MetaCyc
			[M-H]-	CHO	0.952	0.524	447.09286	3.31	[M-H]-	2	kaempferol 3-O-β-D-glucoside	CPD1F-453	GrapeCyc|PlantCyc|MetaCyc
			[M-H]-	CHO	0.952	0.524	447.09286	3.31	[M-H]-	2	kaempferol 7-O-glucoside	CPD-8007	PlantCyc
			[M-H]-	CHO	0.952	0.524	447.09286	3.31	[M-H]-	2	luteolin 4'-O-β-D-glucoside	CPD-25365	MetaCyc
			[M-H]-	CHO	0.952	0.524	447.09286	3.31	[M-H]-	2	luteolin 7-O-β-D-glucoside	LUTEOLIN-7-O-BETA-D-GLUCOSIDE	PlantCyc|MetaCyc
			[M-H]-	CHO	0.952	0.524	447.09286	3.31	[M-H]-	2	orientin	CPD-15083	PlantCyc|MetaCyc
			[M-H]-	CHO	0.952	0.524	447.09286	3.31	[M-H]-	2	quercetin 3-O-rhamnoside	CPD-8013	PlantCyc|MetaCyc
			[M-H]-	CHO	0.952	0.524	447.09286	3.31	[M-H]-	2	quercitrin	QUERCITRIN	MetaCyc
	473.1876649	$C_{18}H_{34}O_{14}$	[M-H]-	CHO	1.889	0.778	473.18677	2.40	[M-H]-	2			
			[M-H]-	CHO	1.889	0.778	473.18675	2.65	[M-H]-	2			
	509.1513470	$C_{20}H_{30}O_{15}$	[M-H]-	CHO	1.500	0.750	509.15057	1.06	[M-H]-	2			
			[M-H]-	CHO	1.500	0.750	509.15063	1.78	[M-H]-	2			
	539.1619911	$C_{21}H_{32}O_{16}$	[M-H]-	CHO	1.524	0.762	539.16118	1.20	[M-H]-	5			
			[M-H]-	CHO	1.524	0.762	539.16141	1.40	[M-H]-	5			
			[M-H]-	CHO	1.524	0.762	539.16141	1.90	[M-H]-	5			
			[M-H]-	CHO	1.524	0.762	539.16132	2.13	[M-H]-	5			
			[M-H]-	CHO	1.524	0.762	539.16111	2.55	[M-H]-	5			
